# Academy of Stomatology

**Published:** 1905-06

**Authors:** 

**Affiliations:** Academy of Stomatology


### ACADEMY OF STOMATOLOGY.

A SPECIAL meeting of the Academy of Stomatology of Philadelphia was held at its rooms, 1731 Chestnut Street, on the evening of Friday, December 9, 1904, to hear a paper by Dr. E. K. Wedelstaedt, of St. Paul, Minn., entitled "Conditions of Failure."

(For Dr. Wedelstaedt's paper, see page 358.)

### DISCUSSION.

*Dr. Edwin T. Darby.*—I do not know that there is very much in the paper with which we can differ, and our discussion must therefore be in the way of agreeing with what Dr. Wedelstaedt has said. There is one point which we all ought to practise, and that is, making a study and record of our conditions of failure. The doctor has gone to a great deal of trouble to show just the conditions which exist before and after operations. It is undoubtedly true that many fillings upon proximal surfaces fail just because the condition represented in Fig. 2 is allowed to prevail. He accounts for that space between the teeth by saying that the operator probably passed a revolving disk or sand-paper disk from the occlusal to the gingival border of the filling. An up-to-date dentist who is retaining contour would have too good judgment to do just that thing.

I have frequently seen it done by students, and it has made a beautiful operation a failure. The disk has been run through and a space left in which food becomes impacted. A disk ought never to be passed between the teeth after the contour has been restored.

I was hoping that the doctor would say something about extension for prevention. It is the greatest advance in the way of saving operations that we have had in the last twenty or more years. I accepted this idea with a good deal of reluctance, but I have been carefully observing the conditions which I have found in the mouths of my own patients as well as in the mouths of patients coming from other dentists, and I have found that the weak point is along the gingival or buccal border near the gum, at the cervix or near the cervix, and that by following the plan illustrated in Model No. 5 better results are accomplished than by our old method of limiting the cut. I have become what might be called a rather excessive advocate of cutting the buccal and lingual border. The method not only simplifies the operation, but prolongs its usefulness as a saver of the teeth. I might not have said this ten or twenty years ago, but I am so thoroughly convinced that that is the proper procedure that I have adopted it in my own practice to the exclusion of any other. It does simplify the packing of gold, the finishing of the filling, and the completion of the whole operation.

*Dr. M. L. Rhein.*—This subject is certainly of great interest to all of us. I more than echo the sentiment of the last speaker upon the value of the extension of cavities in the ultimate salvation of the teeth; but, as I said about two years ago before this Academy, I take decided issue with the statement that it is of recent origin or that it is new within the past twenty-five years. I base my assertion, as on that evening, upon the illustrations in Webb's work on "Operative Dentistry." I can never let an opportunity of this kind pass without giving credit to him and to dentists prior to him for advocating this method of preparation, perhaps not with the scientific exactness so well described by Dr. Black, but with all the essential requirements of the case.

There is no opportunity to-night to take issue with the speaker upon his criticism of failing to produce as far as possible a physiological contact point, but I have never listened to anything, either by word of mouth or writing, of Dr. Wedelstaedt's that I have not become extremely antagonistic to his point of view. He speaks at the close of his address about the few men in the Northwest who are ready to make the filling of teeth scientifically exact. As I understand that, he means that he is going to tell the students and the profession the exact measurements for each cavity, the exact amount of gold that shall be placed in each one, and the exact form of contact point. Now, if such a thing were possible I would at once give up operative dentistry, because there would be no attraction in it for me. It would become too commonplace a matter. To me the attraction of saving the human teeth has been in the variance from the normal physiological type. We do not get teeth to fill with normal occlusion and normal points of contact. A dentist's inventive ability is called into play every time he is called upon to fill a tooth so that it may be preserved. The attraction in the work is that when the operation is completed it differs in some slight or great measure from the one that has preceded it. In the majority of cases coming to your office you cannot make such points of contact as are illustrated here. This is the point which I am anxious to bring out in this discussion. Regarding Model No. 5, showing a double compound filling of mesial, distal, and occlusal surfaces, there is no statement made of the amount of tooth-structure destroyed by caries. I have seen teeth filled in the way here shown as correct utterly unable to withstand the force of mastication. This is to me not a proper criticism to make on what he has shown, because I have no doubt that if that tooth were sufficiently destroyed by caries, Dr. Wedelstaedt would have cut down the cusps of the lingual and buccal sides and replaced them by filling-material, so that they would be protected. It illustrates, however, the main point of my criticism, in showing how unjust it is to judge every man's failures in the scarcely ethical way we have listened to this evening. In some mouths there are such irregularities that it is impossible to separate the teeth as he advocates should be done and get the proper results.

*Dr. S. H. Guilford.*—This method of separating, contouring, and finishing the fillings as described by Dr. Wedelstaedt has been carried on in this section of the country for a very long while; it has also been regularly taught in the colleges.

A matter of almost equal importance to that of preservation of the teeth is the consideration of comfort to the patient. Patients who have had teeth cut, mutilated, and crowded together have been annoyed beyond measure by the discomfort which they have had to endure. All of us have met with such cases, and have noticed how grateful patients are after those conditions have been changed. I remember distinctly a case referred to me by Dr. Jack many years ago. The patient was a man of thirty, and had travelled abroad a great deal. I found upon examination that he had the full complement of teeth above and below, but that they had been filled and refilled and filled again with various filling-materials. He had in the course of his travels fallen into the hands of many dentists, and his teeth had been cut in such a manner that they pressed flatly against each other. I saw that an enormous amount of work would be necessary, and told him it was a question of his willingness to undergo so much repair. He expressed his willingness to endure anything if satisfactory results could be obtained. He went through the tedious operations without a murmur, and from that day to this he has had entire comfort. I never have done such an amount of work in any one mouth, either before or since. His teeth are good in structure, and having been put into their original form, very little has had to be done from that time to this.

Dr. Wedelstaedt and those associated with him are enthusiasts upon this subject, and we need men of that kind in order to awaken a certain amount of energy in the younger men. Dr. Wedelstaedt is not a college teacher, but he is a teacher, nevertheless, and he and his society are doing good work. I believe that in the near future we shall have a school of operators in the Northwest superior to the same number in the Eastern States. The doctor is doing good work and getting others to do good work, and in that respect it seems to me that he is an ideal teacher. I wish to thank him to-night for his paper, and especially for what he has done in his locality.

*Dr. William H. Trueman.*—While I have listened with a great deal of interest to the paper of this evening, and it has been a pleasure to hear Dr. Wedelstaedt explain his ideas in regard to the cause of failures and his method of making a permanent record of the conditions leading to, and resulting from such failures, I am not much impressed with their value. These plaster models which he presents may faithfully depict the conditions he observed prior to taking the impressions from which they have been made, but without a full history of each particular case they will often be misleading and unjust to the operators whose work is thus presented for criticism. A few days ago I replaced a filling that would have made a capital illustration of a failure from the essayist's stand-point; but when I tell you that a few months ago it was in excellent order, and that the dentist who inserted it has been in his grave over thirty years, can you, gentlemen, consider it a failure? I recall another case. The models and the diagrams before you would serve very well to illustrate it, so nearly are they like it. It was a flat gold filling in the posterior approximal surface of an upper second bicuspid. No attempt had been made to contour or to make a contact point; the bicuspid and molar came together, making an ideal place for recurring decay according to present notions. When I first saw it, it had been in place a little over fifty years, and was in perfect order. A few years later I found it necessary to patch it with gutta-percha. Now, a model of that case might have presented some points for criticism, but, remember, the model tells but one part of the story. In this case, no matter how accurately the model would have represented the condition of that filling and its surroundings, it would not have told you that the patient carried it with her to her grave over sixty years after Dr. Edward Hudson had inserted it, and that, permit me to suggest, is important in considering the question whether or not that filling was a failure.

Any filling which has fully performed its office sixty years, or for thirty years, in many cases ten years, or even five years, is not to be lightly spoken of. It may not conform to our notions of what should have been, but who can say that our method would have done better? We strive for permanent work, but "permanent" means a very long time. I am strongly impressed that some methods designed to insure permanency invite destruction. There is no one cause of failure, and no one method of preventing it. I meet with some dentures I cannot save. Whether the cause is in the teeth, or is due to their surroundings, I cannot tell—I set it down to predestination. Now and again such cases fall into the hands of operators who see my mistakes and try to do better, and not unfrequently the poor patient gets a third denture a few years sooner as the result. Many are born into the world badly handicapped, and many of these will live longer and enjoy life more by gracefully accepting the handicap and making the best of it.

The essayist bases his hope of better results by adopting a method different from that which he contends has invited failure in the cases illustrated, on the presumption that the present theory of dental caries is true. Now, do we know beyond doubt the cause of dental caries? We thought we did a great many years ago, but there has been a continual change. A theory lasts about a generation, it is then discarded for a new one more plausible or more scientific. We can do no more when filling an approximal cavity in a biscuspid, by contouring, by properly placing and shaping the contact point, and by extension for prevention, than to approximately make it as safe from germ intrusion as is a filling upon a buccal or labial surface. A well-placed filling upon a buccal or a labial surface is ideal for perfect technic and perfect cleanliness, and yet, nevertheless, recurring decay at the margins of these fillings is not unknown. Why is it that a slight defect in an approximal gold filling is so disastrous, while a filling of gutta-percha in the same position may be imperfect, unclean, an ideal germ culture bed, and yet perfectly protect the tooth from decay. Gutta-percha is not a germicide. There is one thing that I admire very much in Dr. Miller. He has done a great deal of earnest work in the attempt to solve this perplexing problem. His conclusions have more nearly met observed conditions than have those of his predecessors, and none, perhaps, has been so well qualified to pronounce a positive opinion than he, and yet, I observe, he always stops a little short of being absolutely "sure." The last word has not yet been said. Within a few months the question has been raised whether or not a certain constituent of the saliva may be the real destructive agent, or whether it is merely the pabulum that nourishes tooth-destroying germs. The theory of microbic plaques, that a few years ago was accepted as solving one phase of tooth destruction, is now being abandoned.

Who can tell what will be taught the next generation? What becomes of a practice based upon a false theory?

The study of the cause of failures is very interesting; it is important, and, carried on in the right way, is instructive; but I think we should go very, very, *very* slowly in drawing absolute conclusions, because, in a little while the theory on which we are now working may become a back number; there are many things concerning dental caries that it does not explain.

*Dr. M. I. Schamberg.*—Extremists in dentistry are necessary to bring out valuable points whereby we may profit. Dr. Rhein has alluded to the extreme lessons taught by Dr. Wedelstaedt, and takes exception to them. We are all familiar with the immense amount of good Dr. Rhein has done by taking a decided stand in regard to pyorrhœa. I believe that we are ready to admit that a similar good will follow the teachings of Dr. Wedelstaedt in reference to conditions of operative failure.

Yesterday I treated a brother of mine for the condition of failure noted in Fig. 3, where the point of contact of two approximal fillings is near the gingival border. He was suffering very much with a second lower molar tooth which had been carefully examined and no defect in the tooth found. Before taking an X-ray picture, which he desired me to do, I passed an explorer down to find whether there was caries beneath either of the approximating fillings. I found that the fillings were in contact at and were overhanging the gingival margins of the two cavities. The V-shaped space in which food became packed during mastication was causing pericemental trouble. Relief was promptly afforded by taking sand-paper strips and smoothing away the lower portion of two fillings until they approximated nearer the occlusal surface of the teeth. I do not offer this as a method for relieving that condition of failure, but wish merely to call attention to the possibility of the production of pericemental inflammation through the improper filling of teeth. In a case sent to me yesterday by Dr. Fogg the patient was suffering with a lower second bicuspid tooth which, being devitalized, was carefully cleansed by Dr. Fogg and put in a healthy condition for filling. In spite of the good treatment the tooth received, it pained as if it were vital and responded to an application of chloride of ethyl. Dr. Fogg had also noticed that upon the gum there was an enlargement indicating the possibility of root exostosis. An X-ray picture which I took showed a marked enlargement of the roots of this and the adjoining teeth. I advised extraction, and, in spite of the fact that the patient was eighty-two years of age, I gave him gas and removed the tooth, and with it came the tooth immediately in front of it.

The specimen which I pass among you is interesting, and shows that the teeth came out together because of their attachment at the apical end, due to the hypercementosed condition of both teeth. The unusual phenomenon in this case was the transmission of the sensitivity of the first bicuspid to the second bicuspid through the attachment at their apices. In a large number of cases I believe the packing of food between teeth to be the cause of pericemental troubles. In my search for other causes for exostosis I find and am willing to accept this as the most frequent etiological factor in the production of this abnormality.

*Dr. G. Milliken.*—There is little to say in discussion upon this system of Dr. Wedelstaedt. We were always taught it at the University of Pennsylvania, and we have insisted upon the contouring of teeth to a greater or less degree, extending the borders out to the brush line. In the last few years we have been teaching extension for prevention; but as to leaving spaces such as Dr. Wedelstaedt describes, this is foreign to any teaching that I have ever heard at the University of Pennsylvania.

*Dr. G. W. Warren.*—I shall not attempt to discuss in detail the paper of the evening, the ground having been fully covered by Drs. Darby, Guilford, and Trueman. It is true that the principle set forth in the essay—that in filling teeth they should, as far as possible, be restored to their normal physiological condition—has for many years been taught in our colleges. It is equally true that young practitioners, after graduating and getting away from the guidance of their instructors, fall into slipshod methods of restoration; hence the necessity for reiterating these basal facts in our literature and societies. I am glad of the opportunity to thank Dr. Wedelstaedt for his essay and for coming so far to present it. Many good things are coming from the Northwest.

I was interested in Dr. Trueman's remarks as to just what operations we might call failures; to my mind the term is a relative one. Shall we consider the filling which has preserved a tooth for ten, fifteen, or twenty years a failure? This calls to mind an incident of practice occurring only a short time ago. An old gentleman called my attention to a filling on the distal surface of a lower bicuspid tooth; upon examination I found it needed a slight repair. The gentleman then said, "I want to tell you that your father placed that filling there thirty-two years ago." Here was an operation which had been serviceable for over thirty years, shall we call it a failure, then, because the tooth breaks down around it?

Dr. Trueman, in his remarks, also alluded to the several theories as to the cause of dental decay which from time to time have been advanced and later shown to be erroneous, and he cautions us not to accept too fully the present Miller theory, lest it, too, in time be disproved. I cannot agree with the doctor on this point, for, as I view it, we are accepting the present germ theory as conclusive, not because Dr. Miller has advanced the theory, but because he has demonstrated that the principal and basal cause of dental decay is the growth of oral bacteria.

*Dr. James Truman.*—I, unfortunately, was absent when the paper was read, and I hoped that I might receive some information from the discussion concerning the subject of the paper, but though I have listened intently, I can gather nothing from what has been said as to the topic. It may be my fault.

In other respects, I may say that I have a great deal of faith in the Northwest. I am perfectly willing, therefore, to believe that all Dr. Wedelstaedt has said here this evening is right. As I look at those drawings, my mind goes back forty or fifty years to the time when I was but a youth in dentistry, and when I had something to do with teaching operative dentistry, and I must say that, if they represent the present method of filling advocated, it is done exactly as we extended cavities for prevention at that period. I do not see that there is any really great advance. I may be mistaken, however, as I was not here when the paper was read.

*Dr. M. L. Rhein.*—There is one point which I would like to incorporate in my discussion. In this subject of failures in operative dentistry, as illustrated by the charts here, it struck me that Dr. Wedelstaedt omitted what I consider the most important point of all, and that is the lack of proper specific gravity in the filling-material. I am convinced from my experience of many years that most of the instances of recurrent decay in occluso-approximal cavities is due to the ruin of the filling-material under the stress of mastication caused by the running of the gold rather than from insufficient extension, as described by the disciples of Dr. Wedelstaedt.

So far as I can see, my criticisms of Dr. Wedelstaedt concerning these models were a part of the discussion, and with the exception that I said a word in response to what Dr. Darby said, I confined my remarks entirely to the casts, and spoke entirely to the subject.

Regarding the models, the question is simply one of how you read casts. The actual conditions seen in the patient's mouth, with the history then presented, are much more easily understood than casts which are passed around. The question before us this evening is very similar to that of the radiograph. One man will see one thing, and another man something else.

*Dr. Roberts.*—I would like to ask Dr. Wedelstaedt how long a filling should last when it has been properly prepared to be a success?

*Dr. E. K. Wedelstaedt* (closing).—Do good and be kind, is the motto I try to live up to. I came before the members of this society with something which I supposed to be new, something which I do not say is new, but something which I have never seen before, although I have been in the practice of dentistry nearly thirty years. The whole theory of dentistry, from pyorrhœa alveolaris to the spreading of gold, seems to have been discussed, and with the exception of a few who have said kind words, very little has been said upon the subject. I brought you a method for studying conditions of failure. I said nothing whatever of extension for prevention. We know what abnormal conditions are and the necessity for correcting them. All that I said was that teeth should be brought to their normal positions. I shall not, therefore, discuss extension for prevention. I shall not answer anything about specific gravity of gold. I did not come here for this purpose, and I am surprised that a thing of this kind should come up. I do not think that when I come before you with plaster-of-Paris casts of conditions as I have found them that I am dogmatic. I said nothing about Dr. Black. There are the casts, and they tell the truth. I am glad that there is in the dental profession a little band of earnest men who are coming more and more closely together and who study conditions of failure in the same manner in which I have presented them to-night.

Otto E. Inglis,
*Editor Academy of Stomatology.*

